# Ultra-processed food addiction symptoms profile according to weight status among Brazilian adults

**DOI:** 10.3389/fpsyt.2025.1642630

**Published:** 2025-07-29

**Authors:** André Eduardo Silva Júnior, Mateus de Lima Macena, Déborah Tenório da Costa Paula, Délis Barbosa Soares, Isabelle Rodrigues de Souza Gama, Natália Gomes da Silva Lopes, Maria Eduarda de Carvalho Macário da Silva, Ashley Nicole Gearhardt, Nassib Bezerra Bueno

**Affiliations:** ^1^ Laboratório de Nutrição e Metabolismo (LANUM), Faculdade de Nutrição, Universidade Federal de Alagoas, Maceió, Brazil; ^2^ Postgraduate Program in Nutrition, Escola Paulista de Medicina, Universidade Federal de São Paulo, São Paulo, Brazil; ^3^ Programa Associado de Pós-Graduação em Ciências de Movimento, Instituto de Educação Física e Esporte, Universidade Federal de Alagoas, Maceió, Brazil; ^4^ Department of Psychology, University of Michigan, Ann, Arbor, MI, United States

**Keywords:** eating addiction, behavioral profile, weight status, obesity, food addiction

## Abstract

**Introduction:**

The study aimed to investigate several ultra-processed food (UPF) addiction symptoms according to the weight status and to verify if there are specific symptoms of UPF addiction that differ according to the weight status in adults with a UPF addiction diagnosis.

**Methods:**

This is a cross-sectional study that included adults (18–59 years) of both sexes with UPF addiction diagnosis. Demographic and clinical data were collected, such as body mass index (BMI), diagnosis of depression, and generalized anxiety disorder (GAD). UPF addiction was assessed using the modified Yale Food Addiction Scale 2.0.

**Results:**

In total, 1,074 participants were included. Of this total, 83.3% (n = 895) were female, with a mean age of 23 ± 5 years, of which 36.8% (n = 395) were classified with normal weight, 31.9% (n = 343) with overweight, 19.5% (n = 209) with obesity I, 8.1% (n = 87) with obesity II, and 3.7% (n = 40) with obesity III. The prevalence of UPF addiction symptoms referring to social/interpersonal harm, cut down/quit, role interference, and physical/psychological harm increased progressively with increasing BMI, even after adjusting for age, sex, diagnosis of depression, and GAD.

**Conclusion:**

This study showed a progressive increase in UPF addiction symptom severity with rising BMI levels. Moreover, distinct UPF addiction symptom profiles emerged across various BMI categories. Understanding these nuances can guide the development of targeted interventions and treatment strategies to address this multifaceted behavioral profile effectively. Further research across different populations is imperative to broaden our comprehension of UPF addiction’s impact and expression.

## 1 Introduction

The term ultra-processed food (UPF) addiction, which is defined by the excessive consumption of ultra-processed, hyperpalatable, and, commonly, energetically dense foods with characteristics that can have effects similar to addictive substances, has been drawing the attention of clinicians and researchers dealing with the global public health problem of obesity (1, 2). The latest published systematic reviews evaluating the prevalence of the diagnosis of UPF addiction using the Yale Food Addiction Scale (YFAS) tool have shown high frequencies of this phenomenon, especially in individuals with higher body mass index (BMI), which indicates overweight and obesity (2, 3). As highlighted in the study by Praxedes et al. (2), individuals without weight disorders have a pooled prevalence of UPF addiction of 13%, while individuals with overweight and obesity have 24 and 30%, respectively. Also corroborating these data, a recent systematic review showed that individuals with obesity in the preoperative period for bariatric surgery, with a mean BMI above 40kg/m², have a high prevalence of 32% (4). Such evidence suggests a strong relationship between elevated BMI and a high prevalence of UPF addiction.

Researchers debate this strong relationship between UPF addiction and obesity, but there is a consensus that consuming UPF contributes to the obesity pandemic (5, 6). Although the public health problem of obesity has already been the center of significant research, the resolution of this complex and multifactorial disease does not seem to be close. Part of the problem can be attributed to the food industry, which launches products rich in added sugar, which is considered one of the main components with the highest addictive potential (7–9). Individuals with high UPF addiction symptoms are more prone to fail in response to traditional weight loss treatment approaches, which may be associated with behavioral adaptations similar to the withdrawal and tolerance observed in individuals using addictive substances (5, 10–12).

Despite the clinical relevance of UPF addiction, little research has specifically investigated how this phenotype may exhibit a differential presentation across weight status. While UPF addiction is higher in samples with obesity, most individuals with obesity do not meet UPF addiction diagnosis (2). In addition, some individuals without weight disorders meet the UPF addiction classification and have worse quality-of-life indices in all domains (13). Thus, understanding the UPF addiction phenotype and its presence across different BMI classifications may help develop more targeted treatments to address this behavioral profile. However, we are unaware of any study evaluating the prevalence of each UPF addiction symptom in the different weight statuses (normal weight, overweight, obesity I, II, and III). Given this, the study aimed to investigate the prevalence and number of UPF addiction symptoms according to the weight status and to verify if there are specific symptoms of UPF addiction that differ according to the weight status in adults with UPF addiction.

## 2 Methods

### 2.1 Ethical aspects

The research protocol was approved by the Research Ethics Committee of the Universidade Federal de Alagoas (protocol number 4.410.403). All participants were presented with the Free and Informed Consent Form. After accessing the research link, the participants were given the Free and Informed Consent Form on the online questionnaire’s first page. It was necessary to accept it to access the questionnaire and start data collection.

### 2.2 Study design

This is a cross-sectional study.

### 2.3 Location, sample and recruitment

This study is a secondary analysis of a more extensive study that aimed to determine the prevalence of UPF addiction in Brazilian college students. The study was carried out with college students from 94 public and private universities from all units of the Brazilian federation; more details can be found in da Silva Júnior et al. (14). College students of either sex, aged between 18 and 59 years, who met the criteria for UPF addiction were included. Our study did not include underweight individuals, pregnant women, nursing mothers, or individuals after bariatric surgery. Those who sent incomplete questionnaires and improbable body weight and height values were excluded.

Participants were recruited through invitations sent by e-mail and publications on websites and institutional social networks. The participants knew the research topic (UPF addiction in Brazilian university students) and did not receive any compensation for their participation. Data collection took place between October 27 and December 11, 2020.

### 2.4 Demographic and clinical variables

All participants were asked to provide their age (in years), date of birth, sex, educational institution to which they were linked, and the federative unit in which they resided. In addition, participants reported whether they had received a medical diagnosis of depression.

### 2.5 Economic class

To determine the economic class of the participants, the “Critério de Classificação Econômica Brasil” was used. The instrument considers ownership of goods, the number of bathrooms in the household, education of the head of the family, and access to piped water and paved streets. A score is assigned to each item, which, in the end, is added and can vary from 0 to 100 points. Participants are then classified into one of six possible economic classes: “A” (45–100 points), “B1” (38–44), “B2” (29–37), “C1” (23-28), “C2” (17-22), “D-E” (0-16) (15).

### 2.6 Anthropometry

Body weight (in kilograms) and height (in meters) data were self-reported. The BMI was calculated, and they were classified according to the cutoff points proposed by the World Health Organization (16): underweight (BMI < 18.5 kg/m²), normal weight (between 18.5 and 24.9 kg/m²), overweight (between 25.0 and 29.9 kg/m²), obesity I (between 30.0 and 34.9 kg/m²), obesity II (between 35.0 and 39.9 kg/m²) and obesity III (≥ 40.0 kg/m²).

### 2.7 Generalized anxiety disorder

To identify possible cases of generalized anxiety disorder (GAD), the validated version translated into Brazilian Portuguese of the Generalized Anxiety Disorder 7-item scale (GAD-7) was used (17). The scale consists of seven items that can be answered on a Likert scale with four points ranging from 0 to 3, where 0 represents “never” and 3 “almost every day”. The score is summed and can vary from 0 to 21 points, and participants who reached scores equal to or greater than ten were considered probable cases of GAD based on established cutoffs (18). Cronbach’s α for this sample was 0.90.

### 2.8 Ultra-processed food addiction

The modified Yale Food Addiction Scale 2.0 (mYFAS 2.0) was used to determine UPF addiction (19) in the translated and validated version for Brazilian Portuguese (20). The mYFAS 2.0 consists of 13 items, 11 representing symptoms related to the individual’s eating behavior that refer to aspects of substance use disorders in the DSM-5, and two referring to clinical impairment/impairment. Each question is answered according to the frequency with which it occurs, ranging from “never” to “every day”, each of which has a limit for the criterion of the symptom to be met. In the end, the 11 symptoms are added together to create a symptom count score option. Individuals with two or more symptoms that meet the threshold for any clinical distress/impairment problem are classified as having UPF addiction (21). Cronbach’s α for this sample was 0.88.

### 2.9 Statistical analysis

Categorical variables are presented in absolute and relative frequencies. At the same time, continuous variables are in mean and standard deviation. The ANOVA one-way test was used to test the differences in the number of symptoms between the BMI groups (normal weight, overweight, obesity I, obesity II, and obesity III). Next, a Bonferroni correction was applied to determine which groups presented differences and account for multiple comparisons.

Univariable and multivariable analyses were performed by dividing individuals by BMI classification. Poisson regressions with robust adjustment of variances were used to assess the associations between the prevalences of each symptom of UPF addiction between the different BMI classifications (1 = normal weight; 2 = overweight; 3 = obesity I; 4 = obesity II; 5 = obesity III). Linear regressions were also performed with the mYFAS 2.0 score as the outcome variable. In the multivariable analyses, the variables age (years), sex (1 = male; 2 = female), diagnosis of depression (0 = no; 1 = yes), and GAD (0 = no; 1 = yes) were used as adjustments. An alpha value of 5% was adopted, and all analyses were performed using the statistical software R v.3.6.1, with the aid of the “Rcmdr” and “ggplot2” packages (22).

## 3 Results

**Figure 1:**
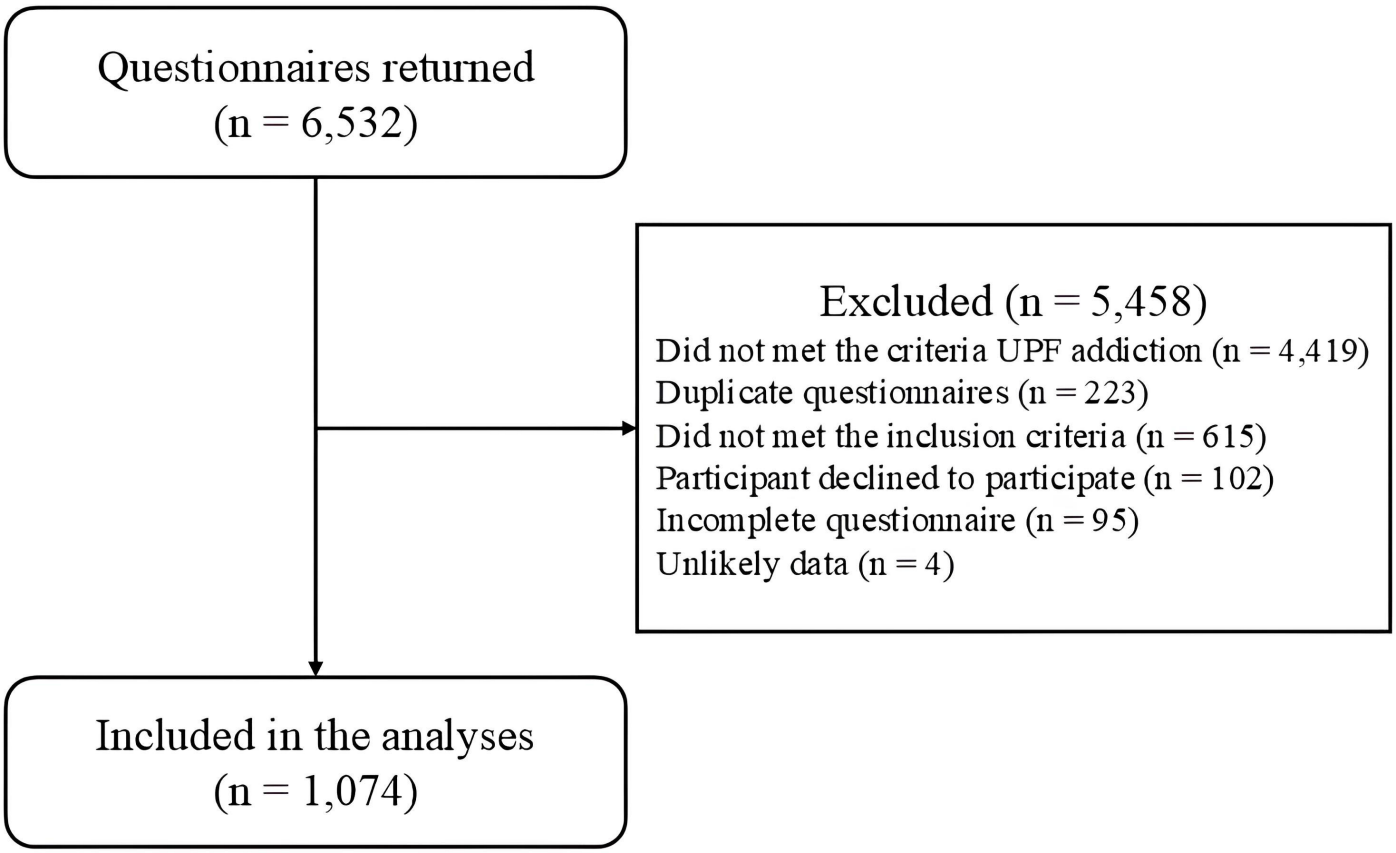
Flowchart of the participants selection.

In total, 6,532 responses were received online; however, after applying the inclusion criteria, data from 1,074 participants were retained in analyses (see **Figure 1** for exclusions). Of this total, 83.3% (n = 895) were female, with a mean age of 23 ± 5 years, of which 36.8% (n = 395) were classified with normal weight, 31,9% (n = 343) with overweight, 19,5% (n = 209) with obesity I, 8.1% (n = 87) with obesity II, and 3.7% (n = 40) with obesity III. The other characteristics of the total sample and according to the BMI classification can be seen in **Table 1**.

**Table 1:** Characteristics of the total sample and divided by body mass index classification.

Caracteristics^a^	Body mass index classification					
	All sample (n = 1,074)	Normal weight (n = 395)	Overweight (n = 343)	Obesity I (n = 209)	Obesity II (n = 87)	Obesity III (n = 40)
	Mean ± SD	Mean ± SD	Mean ± SD	Mean ± SD	Mean ± SD	Mean ± SD
Age (years)	23 ± 5	22 ± 4	23 ± 4	25 ± 6	25 ± 5	27 ± 6
Body mass index (kg/m^2^)	27.8 ± 5.8	22.3 ± 1.7	27.2 ± 1.4	32.2 ± 1.4	36.9 ± 1.3	44.2 ± 3.7
	n (%)	n (%)	n (%)	n (%)	n (%)	n (%)
Sex						
Female	895 (83.3)	339 (85.8)	278 (81.0)	172 (82.3)	72 (82.8)	34 (85.0)
Male	179 (16.7)	56 (14.2)	65 (19.0)	37 (17.7)	15 (17.2)	6 (15.0)
Economic class						
A	149 (13.9)	71 (18.0)	42 (12.2)	26 (12.4)	9 (10.3)	1 (2.3)
B1	154 (14.3)	62 (15.7)	50 (14.6)	30 (14.4)	9 (10.3)	3 (14.8)
B2	338 (31.5)	121 (30.6)	118 (34.4)	57 (27.3)	25 (28.7)	17 (31.8)
C1	241 (22.4)	78 (19.7)	70 (20.4)	55 (26.3)	26 (29.9)	12 (30.7)
C2	138 (12.8)	45 (11.4)	45 (13.1)	29 (13.9)	12 (13.8)	7 (18.2)
D-E	54 (5.0)	18 (4.6)	18 (5.2)	12 (5.7)	6 (6.9)	0 (0.00)
Anxiety	809 (75.3)	308 (78.0)	254 (74.1)	154 (73.7)	60 (69.0)	33 (82.5)
Depression	317 (29.5)	121 (30.6)	93 (27.1)	60 (28.7)	34 (39.1)	9 (22.5)

^a^Categorical variables are presented in absolute and relative frequencies, while continuous variables are in mean and standard deviation. SD, standard deviation.

For individuals with normal weight, the most prevalent UPF addiction symptoms was craving and withdrawal (69.9%, n = 276; 67.1%, n = 265, respectively), while for individuals classified with overweight, the most prevalent symptom was craving (71.7%, n = 246). For participants with obesity I, the most prevalent symptom was physical/psychological harm with prevalences of 75.6% (n = 158), and the participants with obesity II and obesity III, cut down/quit is the most prevalent (80.5%, n = 70 and 87,5%, n = 35, respectively) (**Table 2**). This occurs similarly between the sexes, except for the physical/psychological harm symptom, in which there are no differences between weight status and the male sex (**Supplementary Table S1**). The least prevalent UPF addiction symptoms for participants with normal weight, overweight, and obesity II classifications were used in hazardous situations. Those individuals classified as having obesity I and obesity III indicated giving up activities in response to use as the least prevalent symptom (22.5%, n = 47; 25%, n = 10, respectively).

**Table 2:** Prevalence of symptoms of ultra-processed food addiction among body mass index classifications.

Ultra-processed addiction characteristics^*^	Body mass index classification						
	All sample (n = 1,074)	Normal weight (n = 395)	Overweight (n = 343)	Obesity I (n = 209)	Obesity II (n = 87)	Obesity III (n = 40)	p-value^a^
	Mean ± SD	Mean ± SD	Mean ± SD	Mean ± SD	Mean ± SD	Mean ± SD	
Ultra-processed addiction symptoms	5.9 ± 2.4	5.5 ± 2.4	5.8 ± 2.4	6.2 ± 2.5	6.6 ± 2.2	7.3 ± 2.3	< 0.01
	n (%)	n (%)	n (%)	n (%)	n (%)	n (%)	p-value^b^
Use larger/longer	530 (49.3)	193 (48.9)^a^	163 (47.5)^a^	105 (50.2)^a^	43 (49.4)^a^	26 (65.0)^a^	0.34
Time spent	608 (56.6)	211 (53.4)^a^	189 (55.1)^a^	125 (59.8)^a^	53 (60.9)^a^	30 (75.0)^a^	0.06
Gave up activities	244 (22.7)	86 (21.8)^a^	76 (22.2)^a^	47 (22.5)^a^	25 (28.7)^a^	10 (25.0)^a^	0.70
Withdrawal	735 (68.4)	265 (67.1)^a^	239 (69.7)^a^	140 (67.0)^a^	59 (67.8)^a^	32 (80.0)^a^	0.51
Role interference	600 (55.9)	191 (48.4)^a^	179 (52.2)^a,b^	133 (63.6)^b,c^	65 (74.7)^c^	32 (80.0)^c^	< 0.01
Physical/psychological harm	728 (67.8)	234 (59.2)^a^	235 (68.5)^a,b^	158 (75.6)^b^	67 (77.0)^b^	34 (85.0)^b^	< 0.01
Tolerance	575 (53.5)	224 (56.7)^a^	174 (50.7)^a^	111 (53.1)^a^	45 (51.7)^a^	21 (52.5)^a^	0.58
Craving	770 (71.7)	276 (69.9)^a^	246 (71.7)^a^	150 (71.8)^a^	68 (78.2)^a^	30 (75.0)^a^	0.61
Cut down/quit	739 (68.8)	240 (60.8)^a^	245 (71.4)^b^	149 (71.3)^a,b^	70 (80.5)^b^	35 (87.5)^b^	< 0.01
Hazard	210 (19.6)	66 (16.7)^a^	68 (19.8)^a^	48 (23.0)^a^	16 (18.4)^a^	12 (30.0)^a^	0.16
Social/interpersonal harm	651 (60.6)	199 (50.4)^a^	207 (60.3)^a,b^	143 (68.4)^b,c^	69 (79.3)^c^	33 (82.5)^b,c^	< 0.01

^*^Categorical variables are presented in absolute and relative frequencies, while continuous variables are in mean and standard deviation. ^b^p-value for chi-square test with adjustment by Bonferroni method. Superscript letters characterize a subset of weight status whose proportions show statistically differences. SD, standard deviation.

**Figure 2:**
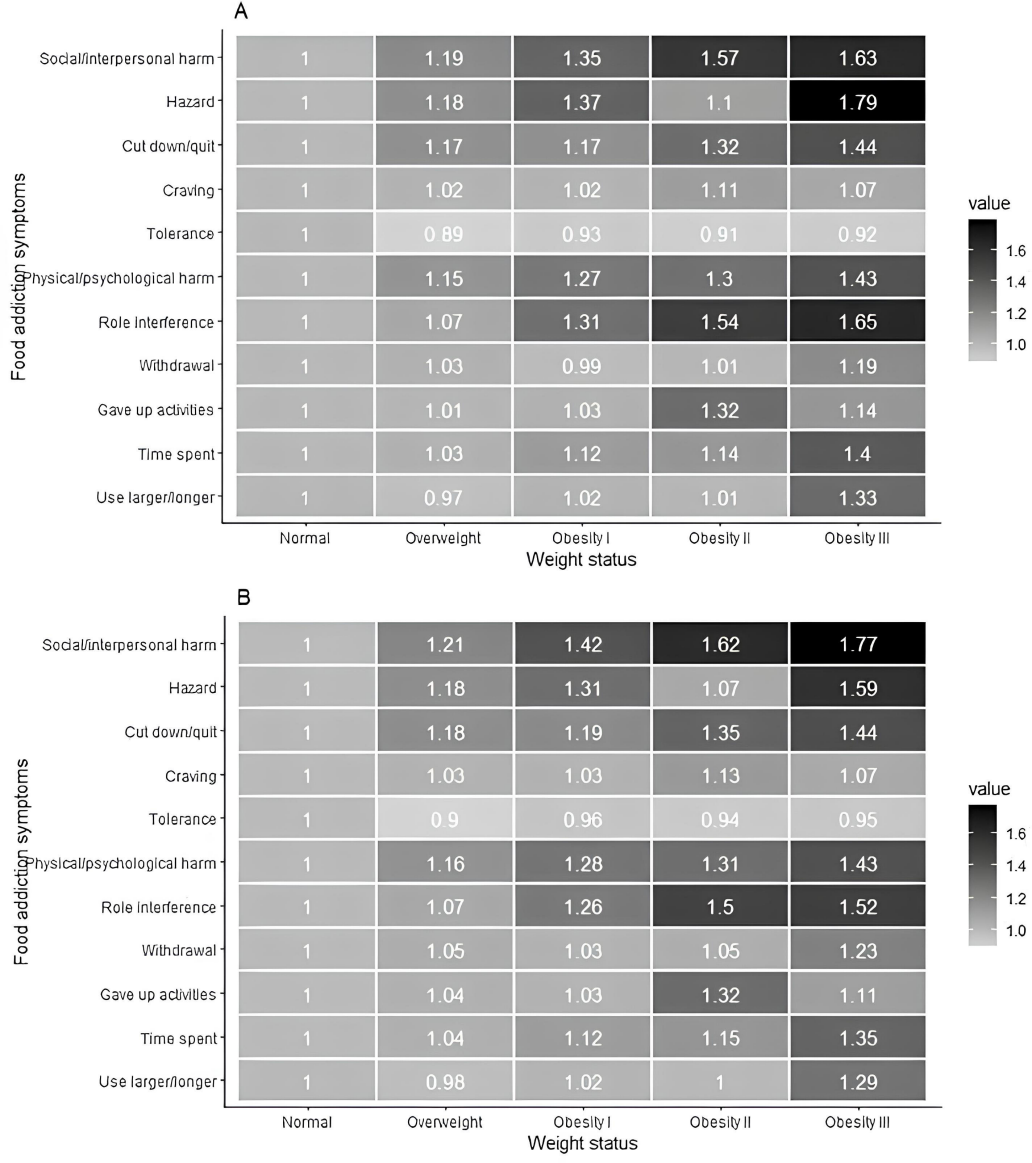
Heatmaps for prevalence ratios from univariable and multivariable analysis between ultra-processed food addiction symptoms and weight status. Footnote: **(A)** Univariable analysis between body mass index ratings and ultra-processed food addiction symptoms. **(B)** Multivariable analysis between body mass index classification and ultra-processed food addiction symptoms. Multivariable analysis adjusting for age, sex, diagnosis of depression and diagnosis of general anxiety disorder. Prevalence ratios by Poisson regression with robust adjustment of variance.

**Figure 2A**, **Supplementary Table S2** show the positive and significant associations found in the univariable analyses between the prevalence of UPF addiction symptoms between the BMI classifications, highlighting the positive associations observed for symptoms of role interference, Physical/psychological harm, cut down/quit, and social/interpersonal harm. All associations remain positive and significant in multivariable analyses with adjustments for age, sex, diagnosis of depression, and diagnosis of GAD (**Figure 2B**, **Supplementary Table S3**).

Univariable and multivariable analyses between UPF addiction symptom numbers and BMI classifications can be seen in **Table 3**. The number of UPF addiction symptoms was significantly different for individuals with overweight relative to the normal weight classification (univariable: β = 0.36; 95%CI: 0.01; 0.71; multivariable: β = 0.42; 95%CI: 0.07; 0.77). Furthermore, individuals in all obesity categories (I, II, and III) were significantly more likely to endorse UPF addiction symptoms than normal-weight individuals.

**Table 3:** Univariable and multivariable analysis between body mass index classification and ultra-processed food addiction symptoms (n = 1,074).

Body mass index classification	Ultra-processed food addiction symptoms			
	Univariable analysis		Multivariable analysis*	
	β	95% CI	β	95% CI
Overweight	0.36	0.01; 0.71	0.42	0.07; 0.77
Obesity I	0.73	0.31; 1.14	0.79	0.37; 1.20
Obesity II	1.13	0.56; 1.70	1.21	0.64; 1.77
Obesity III	1.84	1.04; 2.64	1.82	1.02; 2.61

The normal weight classification was used as a reference category for performing the univariable and multivariable analyses. *Multivariable analyzes adjusting for age, sex, diagnosis of depression and diagnosis of general anxiety disorder.

## 4 Discussion

In this large sample (n = 1,074) of Brazilian college students, it was possible to observe a progressive increase in the number and prevalence of role interference, Physical/psychological harm, cut down/quit, and social/interpersonal harm UPF addiction symptoms as the individuals’ BMI increased. These findings remained after statistical adjustment for age, sex, diagnosis of depression, and GAD. Regarding the number of UPF addiction symptoms, positive associations were observed with obesity classifications I, II, and III, compared to the normal weight group.

The findings that indicate that the prevalence of UPF addiction increases with the increase in BMI corroborate the results of other studies. Praxedes et al. (2) performed an extensive systematic review with meta-analysis. They observed that the prevalence of UPF addiction according to the BMI classification shows a linear growth trend. That is, as the BMI increases, the prevalence of UPF addiction also increases, as also observed in other studies (23, 24). This fact may contribute to the hypothesis that UPF addiction can present as a distinct phenotype within individuals with obesity, which may represent greater susceptibility to the reinforcing effects of highly processed foods (25). Corroborating this, as observed in our study, the magnitude of associations between UPF addiction symptoms and BMI scores appears to be greater at higher degrees of obesity.

In our study, we observed that individuals with normal weight had a high prevalence of the following UPF addiction symptoms: craving, withdrawal, and cut down/quit, while giving up activities and hazards were the least endorsed symptoms. Further, prior research has found that individuals with UPF addiction who are of normal weight also exhibit clinically relevant impairments and may require treatment as well (13). Thus, possible treatments for individuals with UPF addiction may need to be customized according to weight status.

An important aspect that should be considered in this study is the significant increase in the prevalence ratio observed for the UPF addiction symptom role interference as BMI also increases, especially when taking into account the hypothesis that UPF addiction can present as a distinct phenotype within individuals with obesity, which may represent greater susceptibility to the reinforcing effects of highly processed foods in individuals with higher BMI (25). It is known that obesity not only aggravates chronic non-communicable diseases but also plays a negative role in the quality of life and mental health of individuals. People with obesity may experience a lower quality of life, partly explained by the individuals’ mental health deterioration (26). In addition, obesity can decrease the ability to fulfill functions of daily living and, consequently, reduce aspects of overall quality of life (27). Another important point concerns mental health problems (i.e., depression), which appear to have a high prevalence in individuals with obesity and also in those with UPF addiction, so it is possible to observe a greater contribution to the higher role interference as BMI increases (28–30). In addition, another factor that seems to be linked to maladaptive eating behaviors is weight stigmatization, which is characterized by the social devaluation of people due to their body weight, causing social, economic, and psychological consequences (31, 32). The stigmatization of weight among individuals with obesity can also contribute to greater interference in the role as BMI increases (33). Thus, it is clear that the increased prevalence of the symptom “role interference” and “physical/psychological harm” as BMI increases observed in our study is closely linked to the fact that obesity, per se, decreases the quality of life and mental health with repercussions on the functional capacity of these individuals.

It was observed that individuals with overweight and obesity also had a higher prevalence of craving and failed cut down/quit symptoms, as determined by mYFAS 2.0, than individuals with normal weight. This finding is particularly interesting in a population with overweight or obesity, where one of the central intervention goals is to promote weight loss. Individuals with UPF addiction may consume more UPF and less fresh and minimally processed foods (34). Interventions for weight loss usually involve changing the individual’s dietary pattern, increasing the consumption of fresh and whole foods, and decreasing energy-dense foods that individuals commonly identify as addictive (e.g., chocolate, French fries, baked goods) (35). Because of this, the reduction in highly processed consumption may be an agent that hinders adherence to dietary interventions for weight loss in individuals with obesity and UPF addiction, contributing to the high dropout rates evidenced in dietary interventions (11). That said, the ability to control the intake of highly processed foods seems essential for adherence to the dietary treatment of obesity. For individuals, psychobehavioral treatments and pharmacotherapy that focus on reward functioning and craving may be needed to improve weight loss treatment success for individuals with UPF addiction. Further, unsuccessful attempts to reduce highly processed food intake may be related to another symptom of UPF addiction, the characteristic withdrawal symptoms.

Withdrawal syndrome is one of the cornerstones of substance use disorders. It is defined as developing physiological and/or psychological symptoms in response to abstinence or decreased use of an addictive substance (36). Withdrawal syndrome is one of the criteria for diagnosing substance use disorders. In our study, among individuals with obesity III, withdrawal (80.0%) was one of the most endorsed symptoms of UPF addiction assessed by mYFAS 2.0. The prevalence of withdrawal is higher than that of those observed by Hauck et al. (23), in which 14.7% of overweight individuals endorsed the withdrawal criterion. Initial evidence for the plausibility of withdrawal from highly processed foods comes from the observation, in animal models, of physical and psychological symptoms of withdrawal in response to the removal of sucrose from the diet of animals (37, 38). The evidence is still early in humans and is usually measured by the YFAS 2.0 and mYFAS 2.0 items (39). Currently, there is only one tool for operationalizing withdrawal symptoms from highly processed foods, the Highly Processed Food Withdrawal Scale (ProWS) (40), which could be used in future research to investigate the response to dietary change across UPF addiction profiles and BMI classifications.

In the present study, we observed a progressive increase in the prevalence of social/interpersonal harm, and physical/psychological harm symptoms the BMI increased. In individuals with obesity class II and III, this symptom of UPF addiction becomes the most prevalent compared to other BMI classifications. This finding may be related to the stigmatization associated with obesity since the prevalence of this symptom increased as BMI increased. Individuals with obesity are often subjected to negative social stereotypes and consequences, for example, that they are lazy, unmotivated, and lack discipline or willpower, as well as limited employment opportunities, lower wages, and unfair treatment in the workplace (31, 41–43). In this regard, a qualitative study that aimed to assess participants’ interpretation of each YFAS 2.0 item and the fit with the diagnostic criteria for DSM-5 substance use disorders reported that interpersonal problems most commonly occurred when a loved one disapproved of the amount or types of food the participant ate (44). Thus, addressing the deleterious impacts of weight stigma in individuals with both UPF addiction and obesity may be a particularly important future study area.

Future research conducted across different populations, age groups, and geographical regions is essential to broaden our understanding of the impact and expression of UPF addiction. Cultural, social, nutritional, and age-related heterogeneity may influence both the prevalence and the symptomatic profile of this condition, highlighting the need for multicenter and representative investigations. Moreover, studies that take into account weight status, age, and variations in addiction symptoms can support the development of more tailored and effective intervention strategies. Such efforts are crucial for improving diagnostic and therapeutic approaches, as well as for informing public health policies aimed at preventing and managing obesity associated with UPF addiction.

This study has limitations. First, weight and height data were self-reported, which may not accurately represent the actual data of the participants. However, self-reporting body measurements is an efficient strategy for epidemiological studies, considering that there is a high concordance between the measures reported by the participants and those measured, especially in a country with continental dimensions such as Brazil (45). Another limitation concerns the use of a self-reported tool to assess UPF addiction symptoms. However, the tool used (mYFAS 2.0) is based on the criteria proposed by DSM-5 for substance use disorder, is the only validated tool, and is widely used for UPF addiction research around the world. Furthermore, there is evidence that individuals interpret the mYFAS 2.0 items in alignment with the DSM-5 diagnostic criteria for substance use disorders (44). Another limitation is the lack of systematic identification of participants with eating disorders, which are frequent comorbidities in cases of UPF addiction. Not taking these disorders into account may have influenced the results and limits the clinical usefulness of the concept evaluated, especially in contexts of diagnostic overlap. Moreover, it is important to acknowledge that behaviors such as chronic dieting, prolonged caloric restriction, and weight cycling can lead to nutrient deficiencies and mental health impacts that may mimic symptoms of UPF addiction. However, the present study did not include specific measures of dietary restraint, dieting history, or weight cycling, which limits the ability to accurately distinguish symptoms related to UPF addiction from those potentially resulting from restrictive eating behaviors. In addition, when invited to participate in the research, the participants were aware that the study aimed to evaluate UPF addiction, which may have induced the participation of individuals with some identification with the theme. Notably, underweight individuals were excluded from this study. Considering that these individuals may have UPF addiction symptoms associated with comorbid eating disorders, such as binge eating, the absence of this population limits the applicability of the findings to this group (46). Finally, our sample consisted of university students, and the results obtained may need to be more generalizable to other groups.

As for the study’s strengths, we highlight that we reached a large and representative sample of Brazilian college students from all regions of the country and public and private higher education. Therefore, we believe that we were able to capture the economic and ethnic diversity of Brazilian college students in our sample. Additionally, by identifying distinct symptom profiles of UPF addiction according to weight status, our findings contribute valuable insight to tailoring interventions, which is supported by recent longitudinal evidence demonstrating the efficacy of targeted treatments for UPF addiction symptoms over a 12-month period (47). Finally, this study was conducted in Brazil, and further research on UPF addiction in developing countries and Latin America is needed to expand global knowledge about this construct (2, 48).

## 5 Conclusion

The number and prevalence of UPF addiction symptoms presented increase linearly with weight status. The most frequent UPF addiction symptoms endorsed by normal-weight individuals were withdrawal, craving, and failed attempts to cut down/quit. Interestingly, the most prevalent symptoms in individuals with overweight and the degrees of obesity were related to role interference, continued use despite social/interpersonal harms, physical/psychological harms, and cut down/quit. Thus, the present study explored the unique profiles of UPF addiction symptoms in individuals by weight status, which may inform the development of interventions targeting these different behavioral profiles and strategies to improve clinical outcomes. Further research across different populations and geographical regions is imperative to broaden our comprehension of UPF addiction’s impact and expression, as well as to develop interventions considering UPF addiction profile and weight status.

## Data Availability

The raw data supporting the conclusions of this article will be made available by the authors, without undue reservation.
