# Supplementary material for: Ultra-processed food addiction symptoms profile according to weight status among Brazilian adults
Source: Front Psychiatry. 2025 Jul 29;16:1642630. doi: 10.3389/fpsyt.2025.1642630 (PMC12339567; doi:10.3389/fpsyt.2025.1642630)
Supplement: Supplementary file 1 [file Table1.docx]

Supplementary Material

Table S1. Prevalence of symptoms of ultra-processed food addiction among body mass index classifications by sex (n = 1,074).

| Ultra-processed addiction symptoms | Body mass index classification | | | | | | | | | | p-value^a^ | p-value^b^ |
| --- | --- | --- | --- | --- | --- | --- | --- | --- | --- | --- | --- | --- |
|  | Normal weight | | Overweight | | Obesity I | | Obesity II | | Obesity III | |  |  |
|  | Female | Male | Female | Male | Female | Male | Female | Male | Female | Male |  |  |
|  | n (%) | n (%) | n (%) | n (%) | n (%) | n (%) | n (%) | n (%) | n (%) | n (%) |  |  |
| Use larger/longer | 166 (49,0)^a^ | 27  (48,2)^a^ | 134 (48,2)^a^ | 29  (44,6)^a^ | 86 (50,0)^a^ | 19  (51,4)^a^ | 37 (51,4)^a^ | 6 (40,0)^a^ | 22 (64,7)^a^ | 4 (66,7)^a^ | 0,48 | 0,79 |
| Time spent | 177 (52,2)^a^ | 34  (60,7)^a^ | 160  (57,6)^a^ | 29 (44,6)^a^ | 100 (58,1)^a^ | 25 (67,6)^a^ | 45  (62,5)^a^ | 8 (53,3)^a^ | 25 (73,5)^a^ | 5 (83,3)^a^ | 0,09 | 0,09 |
| Gave up activities | 76 (22,4)^a^ | 10 (17,9)^a^ | 68 (24,5)^a^ | 8 (12,3)^a^ | 41 (23,8)^a^ | 6 (16,2)^a^ | 22 (30,6)^a^ | 3 (20,0)^a^ | 8 (23,5)^a^ | 2  (33,3)^a^ | 0,69 | 0,68 |
| Withdrawal | 227  (67,0)^a^ | 38  (67,9)^a^ | 197  (70,9)^a^ | 42  (64,6)^a^ | 119 (69,2) ^a^ | 21  (56,8)^a^ | 49  (68,1)^a^ | 10  (66,7)^a^ | 28  (82,4)^a^ | 4  (66,7)^a^ | 0,41 | 0,86 |
| Role interference | 161 (47,5)^a^ | 30  (53,6)^a,b^ | 153  (55,0)^a,b^ | 26  (40,0)^b^ | 110  (64,0)^b^ | 23  (62,2)^a,b^ | 52 (72,2)^b^ | 13  (86,7)^a^ | 26  (76,5)^b^ | 6  (100,0)^a^ | <0,01 | <0,01 |
| Physical/psychological harm | 200  (59,0)^a^ | 34  (60,7)^a^ | 191 (68,7)^a,b^ | 44  (67,7)^a^ | 134  (77,9)^b^ | 24  (64,9)^a^ | 54  (75,0)^a,b^ | 13  (86,7)^a^ | 28  (82,4)^a,b^ | 6  (100,0)^a^ | <0,01 | 0,15 |
| Tolerance | 191  (56,3)^a^ | 33  (58,9)^a^ | 141  (50,7)^a^ | 33  (50,8)^a^ | 92  (53,5)^a^ | 19  (51,4)^a^ | 34  (47,2)^a^ | 11  (73,3)^a^ | 17  (50,0)^a^ | 4  (66,7)^a^ | 0,52 | 0,50 |
| Craving | 239  (70,5)^a^ | 37  (66,1)^a^ | 206  (74,1)^a^ | 40  (61,5)^a^ | 124  (72,1)^a^ | 26  (70,3)^a^ | 55  (76,4)^a^ | 13  (86,7)^a^ | 26  (76,5)^a^ | 4  (66,7)^a^ | 0,76 | 0,44 |
| Cut down/quit | 214  (63,1)^a^ | 26  (46,4)^a^ | 202  (72,7)^a^ | 43  (66,2)^a^ | 123  (71,5)^a^ | 26  (70,3)^a^ | 57  (79,2)^a^ | 13  (86,7)^a^ | 29  (85,3)^a^ | 6  (100,0)^a^ | <0,01 | <0,01 |
| Hazard | 56  (16,5)^a^ | 10  (17,9)^a^ | 58  (20,9)^a^ | 10  (15,4)^a^ | 41  (23,8)^a^ | 7  (18,9)^a^ | 13  (18,1)^a^ | 3  (20,0)^a^ | 10  (29,4)^a^ | 2  (33,3)^a^ | 0,17 | 0,85 |
| Social/interpersonal harm | 174  (51,3)^a^ | 25  (44,6)^a^ | 165  (59,4)^a,b^ | 42  (64,6)^a,b^ | 113  (65,7)^b,c^ | 30  (81,1)^b^ | 57  (79,2)^c^ | 12  (80,0)^a,b^ | 29  (85,3)^c^ | 4  (66,7)^a,b^ | <0,01 | <0,01 |

^a^p-value for chi-square test with adjustment by Bonferroni method for female. ^b^p-value for chi-square test with adjustment by Bonferroni method for male. Superscript letters characterize a subset of weight status whose proportions show statistically differences.

Table S2. Univariable analysis between body mass index classification and food addiction symptoms (n = 1,074).

| Ultra-processed addiction symptoms | Body mass index classification | | | | | | | |
| --- | --- | --- | --- | --- | --- | --- | --- | --- |
|  | Overweight  (n = 343) | | Obesity I  (n = 209) | | Obesity II  (n = 87) | | Obesity III  (n = 40) | |
|  | PR | 95%CI | PR | 95%CI | PR | 95%CI | PR | 95%CI |
| Use larger/longer | 0.97 | 0.83; 1.13 | 1.02 | 0.86; 1.21 | 1.01 | 0.79; 1.28 | 1.33 | 1,03; 1,70 |
| Time spent | 1.03 | 0.90; 1.17 | 1.12 | 0.96; 1.29 | 1.14 | 0.94; 1.38 | 1.40 | 1.14; 1.71 |
| Gave up activities | 1.01 | 0.75; 1.33 | 1.03 | 0.75; 1.41 | 1.32 | 0.90; 1.93 | 1.14 | 0.65; 2.02 |
| Withdrawal | 1.03 | 0.94; 1.14 | 0.99 | 0.88; 1.12 | 1.01 | 0.86; 1.18 | 1.19 | 1.00; 1.41 |
| Role interference | 1.07 | 0.93; 1.24 | 1.31 | 1.13; 1.52 | 1.54 | 1.31; 1.81 | 1.65 | 1.37; 1.99 |
| Physical/psychological harm | 1.15 | 1.03; 1.28 | 1.27 | 1.14; 1.42 | 1.30 | 1.12; 1.49 | 1.43 | 1.23; 1.67 |
| Tolerance | 0.89 | 0.78; 1.02 | 0.93 | 0.80; 1.09 | 0.91 | 0.73; 1.13 | 0.92 | 0.68; 1.25 |
| Craving | 1.02 | 0.93; 1.12 | 1.02 | 0.92; 1.14 | 1.11 | 0.98; 1.27 | 1.07 | 0.88; 1.29 |
| Cut down/quit | 1.17 | 1.06; 1.30 | 1.17 | 1.04; 1.31 | 1.32 | 1.16; 1.50 | 1.44 | 1.25; 1.65 |
| Hazard | 1.18 | 0.87; 1.61 | 1.37 | 0.98; 1.91 | 1.10 | 0.67; 1.80 | 1.79 | 1,06; 3,02 |
| Social/interpersonal harm | 1.19 | 1.05; 1.36 | 1.35 | 1.18; 1.55 | 1.57 | 1.36; 1.82 | 1.63 | 1.37; 1.94 |

Table S3. Multivariable analysis between body mass index classification and food addiction symptoms (n = 1,074).

| Ultra-processed addiction symptoms | Body mass index classification | | | | | | | |
| --- | --- | --- | --- | --- | --- | --- | --- | --- |
|  | Overweight  (n = 343) | | Obesity I  (n = 209) | | Obesity II  (n = 87) | | Obesity III  (n = 40) | |
|  | PR | 95%CI | PR | 95%CI | PR | 95%CI | PR | 95%CI |
| Use larger/longer | 0.98 | 0.84; 1.14 | 1.02 | 0.86; 1.21 | 1.00 | 0.79; 1.26 | 1.29 | 1.00; 1.67 |
| Time spent | 1.04 | 0.91; 1.18 | 1.12 | 0.96; 1.29 | 1.15 | 0.95; 1.39 | 1.35 | 1.10; 1.67 |
| Gave up activities | 1.04 | 0.79; 1.37 | 1.03 | 0.75; 1.41 | 1.32 | 0.90; 1.94 | 1.11 | 0.61; 2.02 |
| Withdrawal | 1.05 | 0.96; 1.16 | 1.03 | 0.92; 1.16 | 1.05 | 0.89; 1.23 | 1.23 | 1.03; 1.47 |
| Role interference | 1.07 | 0.93; 1.23 | 1.26 | 1.09; 1.46 | 1.50 | 1.28; 1.76 | 1.52 | 1.25; 1.84 |
| Physical/psychological harm | 1.16 | 1.04; 1.30 | 1.28 | 1.15; 1.44 | 1.31 | 1.13; 1.51 | 1.43 | 1.22; 1.68 |
| Tolerance | 0.90 | 0.79; 1.03 | 0.96 | 0.82; 1.13 | 0.94 | 0.76; 1.17 | 0.95 | 0.69; 1.30 |
| Craving | 1.03 | 0.94; 1.13 | 1.03 | 0.93; 1.15 | 1.13 | 0.99; 1.28 | 1.07 | 0.88; 1.30 |
| Cut down/quit | 1.18 | 1.07; 1.31 | 1.19 | 1.06; 1.34 | 1.35 | 1.19; 1.54 | 1.44 | 1.24; 1.67 |
| Hazard | 1.18 | 0.87; 1.61 | 1.31 | 0.93; 1.84 | 1.07 | 0.65; 1.76 | 1.59 | 0.92; 2.74 |
| Social/interpersonal harm | 1.21 | 1.06; 1.38 | 1.42 | 1.24; 1.62 | 1.62 | 1.41; 1.87 | 1.77 | 1.48; 2.12 |

In the multivariable analyses, the variables age (years), sex (1 = male; 2 = female), diagnosis of depression (0 = no; 1 = yes), and GAD (0 = no; 1 = yes) were used as adjustments.
